# Ameliorative Effect of Hexane Extract of *Phalaris canariensis* on High Fat Diet-Induced Obese and Streptozotocin-Induced Diabetic Mice

**DOI:** 10.1155/2014/145901

**Published:** 2014-01-09

**Authors:** Rosa Martha Perez Gutierrez, Diana Madrigales Ahuatzi, Maria del Carmen Horcacitas, Efren Garcia Baez, Teresa Cruz Victoria, Jose Maria Mota-Flores

**Affiliations:** ^1^Laboratorio de Investigación de Productos Naturales, Escuela Superior de Ingenieria Quimica e Industrias Extractivas, Instituto Politecnico Nacional, Avenida Instituto Politécnico Nacional S/N, Unidad Profesional Adolfo Lopez Mateos, 07708 Mexico D.F., Mexico; ^2^Departamento de Alimentos, Escuela Nacional de Ciencias Biologicas, IPN Carpio S/N, 11340 Mexico D.F., Mexico; ^3^Departamento de Biotecnología y Bioingenieria, CINVESTAV, Avenida IPN S/N, 07360 Mexico D.F., Mexico; ^4^Departamento de Ciencias, Basicas Unidad Profesional de Biotecnologia IPN, Avenida Acueducto S/N, 07340 Mexico D.F., Mexico

## Abstract

Obesity is one of the major factors to increase various disorders like diabetes. The present paper emphasizes study related to the antiobesity effect of *Phalaris canariensis* seeds hexane extract (Al-H) in high-fat diet- (HFD-) induced obese CD1 mice and in streptozotocin-induced mild diabetic (MD) and severely diabetic (SD) mice.AL-H was orally administered to MD and SD mice at a dose of 400 mg/kg once a day for 30 days, and a set of biochemical parameters were studied: glucose, cholesterol, triglycerides, lipid peroxidation, liver and muscle glycogen, ALP, SGOT, SGPT, glucose-6-phosphatase, glucokinase, hexokinase, SOD, CAT, GSH, GPX activities, and the effect on insulin level. HS-H significantly reduced the intake of food and water and body weight loss as well as levels of blood glucose, serum cholesterol, triglyceride, lipoprotein, oxidative stress, showed a protective hepatic effect, and increased HDL-cholesterol, serum insulin in diabetic mice. The mice fed on the high-fat diet and treated with AL-H showed inhibitory activity on the lipid metabolism decreasing body weight and weight of the liver and visceral adipose tissues and cholesterol and triglycerides in the liver. We conclude that AL-H can efficiently reduce serum glucose and inhibit insulin resistance, lipid abnormalities, and oxidative stress in MD and SD mice. Our results demonstrate an antiobesity effect reducing lipid droplet accumulation in the liver, indicating that its therapeutic properties may be due to the interaction plant components soluble in the hexane extract, with any of the multiple targets involved in obesity and diabetes pathogenesis.

## 1. Introduction

Obesity is a metabolic disease of pandemic proportions largely arising from positive energy balance, a consequence of sedentary life style, conditioned by environmental and genetic factors [1]. Obesity results from an imbalance between energy intake and expenditure. It is often associated with chronic diseases such as hyperlipidemia, hvpertension and noninsulin- dependent diabetes mellitus and with increased risk of coronary heart diseases [2]. It has been reported that variations in total energy intake and diet composition are important in the regulation of metabolic processes [3]. Excessive accumulation of lipids in nonadipose tissues such as liver, heart, skeletal muscle, kidney, and pancreas contributes to the pathogenesis of fatty liver, heart failure, and insulin resistance with the so-called lipotoxicity mechanism, fatty liver is an early hallmark of nonalcoholic fatty liver disease [4], the most common chronic liver disease associated with insulin resistance, obesity and type 2 diabetes [5].

Adipose tissue, besides its primary function of fat storage, also regulates appetite and metabolism by secretion of certain chemicals like an endocrine gland [6]. Thus, persistent insulin resistance and hyperglycemia lead to the development of diabetes mellitus type 2 (T2DM) [7]. Furthermore, the metabolic syndrome develops atherosclerotic diseases, whose fatality is very high, when the symptom gets worse [8]. It is therefore important to prevent or abate obesity. A growing number of enzymes involved in lipid metabolic pathways are being identified and characterized. They represent a rich pool of potential therapeutic targets for obesity [9]. Triglyceride (TG) metabolism is regulated by several factors such as TG intake from food and synthesis and oxidation in various tissues. Among these factors, the hydrolysis of TG by lipoprotein lipase to free fatty acids is an important determinant in TG metabolism [10]. Canary seed is solely used as food for caged and wild birds. However, the unique composition and characteristics of canary seed make it a promising cereal for food and industrial uses [11]. In**  **the past, canary seed was not seen as a viable cereal for human consumption due to the harmful effects associated with the siliceous hairs that cover the hull of the seed. These hairs are highly irritating when they come in contact with human skin or lungs and have been linked to esophageal cancer [12]. In 1997 CDC Maria was registered in Canada as the first hairless cultivar eliminating the potential health risk associated with hairy varieties [13]. The variety was developed based on mutagenesis and traditional breeding by which a totally hairless variety was developed. Removing the damaging hairs rediscovered canary seed as a potential food crop and industrial crop for fractionation industry.

The canary seed or alpiste, *Phalaris canariensis *L., is a member of a family of grasses (Graminaceae), and it is used in folk medicine in the form of tea as a coadjuvant in the treatment of hypertension, diabetes mellitus, and hypercholesterolemia [14], with or without other forms of traditional therapy [15]; however, such use has no scientific basis. There is only one study related to the hypotensive effect of *P. canariensis* seed infusion in normotensive rats [16]; however, its therapeutic use as an antihypertensive agent and its possible mechanisms of action have not been scientifically demonstrated. The aim of the present study was to establish antiobesity and antidiabetic activities of the seeds of *Phalaris canariensis*.

## 2. Materials and Methods

### 2.1. Plant Material

Seeds of *P. canariensis* were collected in Morelos State, Mexico. A voucher specimen (number 8054) was deposited in the Herbarium of the National School of Biological Sciences, for further reference.

### 2.2. Preparation of Plant Extracts

A total of 1000 g of the seeds of *P. canariensis* were dried and powdered in a mechanical grinder. The grinded material was extracted with 5 L of hexane, chloroform, and methanol consecutively using a soxhlet apparatus. These extracts were filtered and concentrated by a rotary vacuum evaporator and kept in a vacuum desiccator for complete solvent removal.

### 2.3. Animals

The study was conducted in CD1 mice of both sexes, weighing about 25–30 g. Before and during the experiment, animals were fed a standard laboratory diet (Mouse Chow 5015, Purina) with free access to water. Mice were procured from the bioterium of ENCB and were housed in microloan boxes in a controlled environment (temperature 25 ± 2°C). Animals were acclimatized for a period of three days in their new environment before the initiation of experiment. Litter in cages was renewed three times a week to ensure hygiene and maximum comfort for animals. The experiments reported in this study were carried following the guidelines stated in Principles of Laboratory Animal Care National Institute of Health publication (NIH) publication 85-23, revised 1985 and the Mexican Official Normativity (NOM-062-Z00-1999). All animals procedures were performed in accordance with the recommendations for the care and use of laboratory animals (756/lab/ENCB).

### 2.4. Mouse Model of Diet-Induced Obesity

Thirty-five male CD1 mice, 2 months old and weighing between 20 and 25 g, were acclimatized under room temperature (28 ± 2°C) with a regular light/dark cycle and free access to food and water for 1 week before use. Following acclimatization, the animals were randomly segregated into five groups of seven rats each. We formulated experimental high-fat diets consisting of 50% normal rat chow pellet, 24% corn oil, 20% full-cream milk powder, and 6% sugar, as suggested by Martinello et al. [17]. Another group, noted as normal control (NC), was given normal rat chow. After the induction period, the mean body weights of the high-fat diet-treated groups were compared with the NC group. Groups with significantly higher mean body weights than the NC group were considered to be obese and were used in the subsequent 10 weeks of experimentation with *P. canariensis* extracts. Each extract at dose 400 mg/kg was prepared by dissolving in 1% of Tween 80. The supplementations were given daily via oral gavage for 10 weeks, at a volume of 0.1 mL. The NC and obese control (OC) groups were given distilled water as a placebo.

### 2.5. Supplementation of *P. canariensis*

Treatment with extracts began after 10 weeks of obesity induction and scored until week 10. Two different doses of *P. canariensis *were used in the experiment: 200 and 400 mg/kg. The extracts were prepared by dissolving in 1% of Tween 80. The supplementations were given daily via oral gavage for 10 weeks, at a volume of 0.1 mL. The NC and obese control (OC) groups were given distilled water as a placebo. Contrary from the induction period, all groups were given normal rat chow throughout the treatment period. 

### 2.6. Induction of Severe Diabetes (SD)

Severe diabetes was induced in overnight fasting male mice by a single intraperitoneal injection of 50 mg/kg of streptozotocin in a volume 1 ml/kg body weight dissolved in cold citrate buffer (pH 4.5) [18]. Hyperglycemia was confirmed by measuring glucose 72 h after the streptozotocin shot and 7 days after injection, confirming a high glucose level. Mice with permanent high fasting blood glucose level >300 mg/dl were included in the experiments.

### 2.7. Induction of Mild Diabetes (MD)

Mild diabetes was induced in overnight fasting mice by administering a single intraperitoneal injection of 60 mg/kg *b.w. *STZ in 0.1 mol/L cold citrate buffer (pH 4.5), 15 min after the intraperitoneal administration of 120 mg/kg nicotinamide. The STZ treated animals were allowed to drink 5% glucose solution overnight to overcome drug induced hypoglycemia. After 10 days of development of diabetes, mice with moderate diabetes having persistent glycosuria and hyperglycaemia (blood glucose > 250 mg/dl) were used for further experimentation [19].

### 2.8. Experimental Design in Diabetic Mice

#### 2.8.1. Effect of Single Oral Administration of Extracts of *P. canariensis* in Glucose Level in Severe and Mild Diabetic Mice

After the mice had been denied access to food/water overnight, they were randomly divided into eight groups (six mice per group) matched for body weight. Normal mice administered distilled water daily for 30 days. Diabetic control mice administered distilled water daily for 30 days. Diabetic mice administered extract of hexane (100 mg/kg), for 30 days. Diabetic mice administered extract of hexane (200 mg/kg), for 30 days. Diabetic mice administered extract of hexane (400 mg/kg), for 30 days. The other diabetic groups were orally administered 100, 200 and 400 mg/kg body weight (b.w.) of extracts of chloroform (AL-C) and methanol (AL-M) suspended in Tween 80, 1% via gavage). Diabetic treated mice received glibenclamide (GB) a dose of 5 mg/kg b.w as standard drug. Blood samples were collected from the tail vein at 0, 2, 4, 6, 8, and 12 h after the administration. The plasma glucose concentration was determined by an enzymatic colorimetric method using a commercial kit (Sigma Aldrich, USA).

#### 2.8.2. Antidiabetic Test in Chronic Severe and Mild Streptozotocin-Induced Diabetic Mice

In a parallel study eleven groups (*n* = 10) of diabetic mice were used to determine the chronic effect of AL-H extract. Each group was submitted to a specific treatment as follows. Normal control and severe and mild diabetic mice groups were fed with normal diet and drinking water *ad libitum* and were given saline by gastric gavage. Severe and mild diabetic mice that received Al-H extract by gastric gavage (400 mg per kg of body weight) every day [7] were designated as SD + Al-H and MD + AL-H groups. Two groups with severe (SD+GB) and mild diabetes (MD+GB) mice were administered with glibenclamide (GB) 4 mg/kg as positive control.

#### 2.8.3. Determination of Body Weight and Food Intake

The final body weight showed significant increase from the initial body weight in all the groups except in the diabetic group, in which there was significant decrease in body weight compared to the initial body weight (Table 5).

The body weight of each mouse was measured once each week and the total amount of food consumed was recorded 3 times per week.

#### 2.8.4. Collection of Organ Tissues

At the end of obesity and chronic diabetes experiments all mice were anesthetized with 1.0% pentobarbital sodium and blood was obtained from the retroorbital plexus of each animal following the injection of heparin (100 IU kg^−1^ body weight) into a tail vein for 10 min [20]. The liver and kidney were removed according to defined anatomical landmarks [21].

#### 2.8.5. Plasma Biochemical Analysis

Blood samples were collected from tail vein of the mice into microcentrifuge tubes containing heparin (10 *μ*L, 1000 IU mL^−1^). The blood samples were then centrifuged at 1600 ×g for 15 min at 4°C for the preparation of plasma. Concentrations of plasma glucose, total cholesterol (TC), triglycerides (TG), and HDL-cholesterol were measured with enzymatic assay kit (Genzyme Diagnostics), and LDL-cholesterol was calculated as the remaining difference of total cholesterol and HDL. Blood glucose levels were measured employing the glucose oxidase-peroxidase (GOD-POD) method [22]. Lipid peroxidation, that is, thiobarbituric acid reactive substances (TBARS), was estimated by the method of Fraga et al. [23] and expressed as *μ*M/g of liver and kidney tissue. Serum glutamate oxaloacetate transaminase (SGOT), glutamate pyruvate transaminase (SGPT), serum alkaline phosphatase (SALP), and total protein, using a commercial Diagnostic Kit Biocompare, BioVision, Biocompare and Thermo scientific, respectively. Malondialdehyde (MDA) as thiobarbituric acid reactive substances was measured at 532 nm spectrophotometrically [24].

#### 2.8.6. Oral Glucose Tolerance Test

Mice of each group were orally administered *P. canariensis* aqueous extracts at doses of 400 mg/kg body weight on a daily basis for 30 days. At the end of the experiment, an oral glucose tolerance test (OGTT) was performed to assess the animals' sensitivity to a high glucose load. Overnight fasting mice were fed orally 2 g glucose/kg b.w. Blood samples were collected from the caudal vein from a small incision at the end of the tail at 0 min (immediately after glucose load), 30, 60, 90, and 120 min after glucose administration.

#### 2.8.7. Assay of Glycogen Content in Liver and Skeletal Muscle

Mice were  sacrificed by decapitation; livers and kidneys were extracted. Frozen livers were thawed, weighed, and homogenized in Tris-HCl (5 mmol/L, pH 7.4) buffer containing 2 mmol/L EDTA. Homogenates were centrifuged at 1000 ×g for 15 min at 4°C. The supernatant was mixed with glucose-6-phosphate dehydrogenase and NADPH and the activity of hexokinase was determined at 37°C for 3 min at 30 s intervals at 340 nm [25] The hepatic glycogen content was measured according to the anthrone-H_2_SO_4_ method [26]. Briefly, liver tissue (64–144 mg) was homogenized in five volumes of an ice-cold 30% KOH solution and the homogenate was placed in a boiling water bath (100°C) for 20 min. The glycogen was redissolved in distilled water and treated with an anthrone reagent (2 g anthrone/L of 95% (v/v) H_2_SO_4_) and the absorbance was measured at 620 nm.

#### 2.8.8. Assay of G6Pase Activity in Liver

The hepatic G6Pase (glucose-6-phosphatase) activity was assayed by the method of Baginiski et al. [27]. Shortly, the glucose-6-phosphate in the liver extract was converted into glucose and inorganic phosphate. The inorganic phosphate liberated was determined with ammonium molybdate; ascorbic acid was used as the reducing agent. Excess molybdate was removed by the arsenite-citrate reagent, so that it could no longer react with other phosphate esters or with inorganic phosphate formed by acid hydrolysis of the substrate. The amount of phosphate liberated per time unit, determined as the blue phosphor-molybdous complex at 700 nm, was a measure of the glucose-6-phosphatase activity. The protein content of the liver extract was quantified by Bradford reaction (Bio-Rad protein assay kit) [28]. The G6Pase activity (mU) was expressed as mmol of phosphate released/min/mg of protein. 

#### 2.8.9. Assay GK Activity in Liver

Glucokinase (GK) activity was measured using a spectrophotometric method as previously described by Panserat et al. [29]. Briefly, liver tissues were homogenized and the supernatant obtained by centrifugation was supplemented with 1 mM NADP, 5 mM ATP, and 100 or 0.5 mM glucose at pH 7.5. The enzymatic reaction was started by the addition of 0.2 unit of glucose-6-phosphate dehydrogenase (E.C.l.l.l.49) and incubated for 5 min at 37°C. NADPH generated by GK was measured using a spectrophotometer at 340 nm. GK activity was estimated by the standard method, that is, subtracting the rate of NADPH formation in the presence of 0.5 mM glucose from that obtained in the presence of 100 mM glucose [30]. Protein concentration was quantified by Bradford and one unit of enzyme activity (mU) was defined as mmol of substrate molecules converted by 1 mg protein per minute. GK activity was estimated as the difference in activity when samples were assayed at 100 mmol/L (GK plus hexokinase activity) and 0.5 mmol/L glucose (hexokinase activity).

#### 2.8.10. Measurement of Antioxidant and Lipid Peroxidation Parameters

After 30 days of treatment, mice fasted overnight and euthanized by anesthesia. Antioxidant enzyme activities in the liver, pancreas, and kidney were assayed using commercial kits: superoxide dismutase (SOD) assay kit Bioxytech SOD-525 for SOD activity (Oxis International), catalase assay kit for catalase activity (CAT) (Cayman Chemical), glutathione reductase (GSH**)** assay kit Bioxytech GR-340 for GR activity (Oxis International), glutathione peroxidase (GPx**)** assay kit GPx-340 for GPx (Oxis International), and lipid peroxidation using malondialdehyde level by commercial kit (TBARS assay kit). In the pancreas the protein concentration was determined by the Bradford method as described in the Bio-Rad protein assay kit.

#### 2.8.11. Extraction of Hepatic Lipids

After removal from the animals, parts of the samples of fresh liver were collected for analyzing the lipid content. The liver (1.25 g) was homogenized with chloroform/methanol (1 : 2, 3.75 mL), and then chloroform (1.25 mL) and distilled water (1.25 mL) were added to the homogenate and mixed well. After centrifugation (1500 ×g for 10 min), the lower clear organic phase solution was transferred into a new glass tube and then lyophilized. The lyophilized powder was dissolved in chloroform/methanol (1 : 2) as the hepatic lipid extracts and stored at −20°C for less than 3 days [31]. The hepatic cholesterol and triglycerides in the lipid extracts were analyzed with the diagnostic kits used for the plasma analysis.

#### 2.8.12. Determination of Insulin

Diabetic mice serum and pancreatic insulin were measured by a Glazyme Insulin-EIA Test according to the manufacturer's instructions [32]. Blood samples and pancreas were taken for insulin determination. The level of insulin in serum was expressed in *μ*IU/mL.

#### 2.8.13. Statistical Analysis

Statistical analysis was carried out by using commercially available software SigmaStat 3.5. Values are expressed as mean ± SEM. For multiple comparisons, one-way ANOVA was used followed by Tuckey and Dunnett's test. *P* value < 0.05 was considered to be significant.

## 3. Results

Treatment with chloroform and methanol extracts did not significantly inhibit the rise in blood glucose levels in normal and diabetic mice and in oral glucose tolerance. However, mice fed the high-fat diet treated with hexane, chloroform, and methanol extracts showed a decrease similarly; for this reason it was decided to study only hexane extract due to the fact that it presents hypoglycemic and antiobesity activities. However, chloroform and methanol extracts did not.

### 3.1. Effects of the High-Fat Diet (HFD) on Body Weight and Food Intake

The body weights and food intake are shown in Table 1. No significant difference in food intake was observed throughout the obesity induction period in all groups. Body weight of all groups was significantly increased compared with the control group after 10 weeks of feeding.

### 3.2. Effect of *P. canariensis* Extracts on the Weight of Organs and Adipose Tissue

The mice fed on the high-fat diet for 10 weeks had a significantly higher body weight and significantly heavier visceral adipose tissues than the mice fed on the normal diet. In the mice fed on the high-fat diet, the body weight elevation that took place over the initial 6 weeks on the high-fat diet was significantly reduced (Table 2) and the final weights of the visceral adipose tissues were significantly lower than those in the mice fed on the high-fat diet alone. The body weight reduction was proportionally equal to the reduction in the visceral adipose tissue weight after 10 weeks. The weights of organs and adipose tissue of normal and obese mice are shown in Table 3. The high-fat diet led to significant increases in the weights of the visceral adipose tissues, kidney, and liver compared to the weights measured in the normal diet group. Extracts with high-fat diet significantly reduced the final weights of the liver and adipose tissues compared to the levels measured in the animals fed on the high-fat diet alone. The kidney weight was decreased in the HFD group compared with the LFD group. The HDF caused the elevation of plasma total. The moderate doses (200 mg/kg per day) of hexane, chloroform, and methanol extracts significantly decreased the level of plasma total (26%, 28%, and 25% resp.). The VAT and liver weight were significantly lower with extracts groups compared with the control mice at 10 weeks, while in SAT weight between the groups of mice at the end point of the experimental period showed decrease of 11%, 14%, and 13%, respectively (Table 3).

### 3.3. Effect of the *P. canariensis* Extracts on Lipid Profile and Hepatic Lipid

Table 4 shows the plasma cholesterol, level of the experimental animals. Significant increase in the levels of plasma cholesterol was observed in the HFD fed groups for the initial ten weeks. After treatment the plasma cholesterol was significantly decreased in the treatment to 39% (AL-H) as compared to cholesterol level in HFD-fed control groups. The HFD-fed rats receiving 10 weeks of treatment with *P. canariensis* extracts (200 mg/kg per day) also showed significantly lower values of plasma TG 35%, 44%, and 32% for hexane, chloroform, and methanol extract, respectively, compared with the vehicle-treated counterparts (Table 4). The moderate doses (200 mg/kg per day) of *P. canariensis* extracts of hexane, chloroform, and methanol increased the level of plasma HDL (24%, 27%, and 21% increase resp.). The HFD caused elevation of hepatic TC and TG. All *P. canariensis* extracts decreased the level of hepatic TC (25%, 29%, and 24% reduction, resp.). The moderate doses of *P. canariensis* extracts significantly reduced the TG hepatic (35%, 37%, and 33% reduction, resp.). On the one hand, there were no significant differences in lipid profile and hepatic lipid in all groups. The hepatic tryglicerides levels in the normal group, high-fat diet group, and high-fat + *P. canariensis* extracts (AL-H, AL-C, AL-M) diet group were 86.2 ± 6.7, 84.5 ± 5.8, and 87.3 ± 2.0 mg/g liver, respectively. In addition, plasma tryglicerides levels tended to be reduced in the high-fat diet group because of a decrease in triacylglycerol secretion from the liver.

### 3.4. Lipid Peroxidation and Protein Estimation

MDA, which is the final product of lipid peroxidation, was determined spectrophotometrically. HFD increased the MDA level in plasma when compared with that of the normal group. HFD group treatment with AL extracts showed a significant reduction in MDA levels (Table 4).

### 3.5. Effect of *P. canariensis* Extracts on the Activities of Hepatic Enzymes

As shown in Table 4, AL-H exhibits a hepatoprotective effect *in vivo*, indicated with reduced ALP, SGOT, and SGPT levels. It was found that mice fed with high-fat diet alone developed a high degree of steatosis. The effect of increased liver enzymes levels and the formation of steatosis in the high-fat diet-fed group correlates with an increase of liver weight (Table 3). The administration of AL-H resulted in the prevention of hepatic fatty deposition in hepatocytes at dose of 400 mg/kg.

### 3.6. Effect on Diabetic Mice of the Hexane Extract from *P. canariensis* on Body Weight and Food and Water Intake

During the study period of 4 weeks, body weight and food and water intake of each mouse were recorded daily but data is presented only at day 0 and the end of the experimentation period (Table 5). The body weight, liver and kidney weights of mice from STZ control group (after 15 days) were significantly (*P* < 0.001) decreased when compared with normal control group. The extract at dose of 400 mg/kg b.w. significantly (*P* < 0.001) maintained the body weight and liver and kidney weights toward normal as compared with STZ control.

### 3.7. Serum Biochemical Parameters

Biochemical parameters like SGOT, SGPT, SALP, and proteins in the STZ control group were significantly (*P* < 0.001) elevated as compared with the normal control group. Treatment with**  **AL at the dose of 400 mg/kg b.w. significantly (*P* < 0.001) brought the SGOT, SGPT, SALP, and serum protein toward the normal values. The total protein was found to be significantly (*P* < 0.001) decreased in the STZ controls groups as compared with the normal control group indicating a lower capacity for protein synthesis in the livers of the diabetic animals; the administration of hexane extract in types SD and MD diabetic animals significantly (*P* < 0.001) prevented the loss of total protein content as compared with the STZ control group (Table 6).

### 3.8. Effect of *P. canariensis* Extracts on the Serum Lipid Profile and Hepatic and Renal TBARS

There was a significant elevation in serum triglycerides and total cholesterol and TBARS levels in the liver and kidney of diabetic mice while LDL-cholesterol decreased (Table 7). Daily administration of *P. canariensis* extracts at a dose of 400 mg/kg to diabetic mice for 30 days significantly reduced in severe and mild diabetes total cholesterol and triglycerides by 33%, 45% and 45%, 37%, respectively. TBARS level in diabetic mice also decreased after treatment with the extract, 22.0% and 37% in liver and 28% and 29% in kidney. LDL-cholesterol on the other hand upon treatment also got a decrease. The results also demonstrate a significant control of serum lipid profiles in the *P. canariensis* hexane extract treated diabetic mice, with responses comparable with those of the standard drug.

### 3.9. Effect of *P. canariensis* Extracts on the Fasting Blood Glucose Levels in Normal and Diabetic Mice

The oral administration of AL at doses of 100, 200 mg/kg, and 400 mg/kg produced a significant hypoglycaemic effect in normal fasting mice after 4 h. The most pronounced effect of AL was observed after 6 h (Table 8). The dose at 200 mg/kg reduced the blood glucose level of the normal fasting mice from an initial mean value of 102.48 at the initial time (0 h) to a mean value of 50.76 (50%) at the end of the 6 hrs. Whereas, in the group that received 400 mg/kg body weight of the extract, there was a significant reduction in blood glucose level in fasting normal mice (60%) after 6 h (*P* < 0.05). Oral treatment with glibenclamide (4 mg/kg) caused a light reduction in blood glucose levels. Reduction in the blood glucose level caused by AL-H at all doses is higher than that of standard drug, glibenclamide.

### 3.10. Effect of on Fasting Blood Glucose Levels in STZ-Induced Severe Diabetic Mice and STZ-Nicotinamide-Induced Mildly Diabetic Mice

The antihyperglycemic effect of AL-H on the fasting blood glucose levels in STZ-induced SD and**  **STZ-nicotinamide-induced MD diabetic mice is shown in Table 9. In addition, AL-H given at same concentrations produced significant antihyperglycemic effects (*P* < 0.005) on streptozotocin-induced severe and mildly diabetic mice after 2 weeks of treatment. Treatment of diabetic mice with glibenclamide (4 mg/kg) produced a significant fall in blood glucose after 4 weeks (68%). Hexane extract (AL-H) at 400 mg/kg dose gradually decreased blood glucose level 4 weeks after administration (54% and 52%, resp.). In diabetes mildly, continued glucose lowering effect at the end of the study when compared to diabetic control.

### 3.11. Oral Glucose Tolerance Test

The effect of AL-H on glucose tolerance was determined after the 30 days of treatment. Postprandial blood glucose levels in mice show a significant change after glucose loading, increasing rapidly in all groups of diabetic mice within the first 30 min and remaining high over the next 120 min in diabetic control mice.  Table 11 shows the changes in the levels of blood glucose in normal, diabetic control, and experimental groups after oral administration of glucose (2 g/kg). Oral treatment (400 mg/kg) in diabetic control rats with the hexane extract of seeds of *P. canariensis* produced a significant (*P* < 0.05) reduction of glucose in blood at 120 min, showing a 45% and 47% decrease of glucose in blood in severe and mild diabetes, respectively, compared with diabetic mice (Table 10). These results reflect the efficiency of the extract to control elevated blood glucose levels.

### 3.12. Effect on Hepatic Glucose Regulation Enzyme Activities and Skeletal Glycogen Content

Table 11 shows the effect of the hexane extract on G6Pase, GK, and HK activity and glycogen content of liver and skeletal muscle. Administration of AL-Hat 400 mg/kg body weight increased the content of hepatic glycogen, GK, and HK in diabetic mice while G6Pase decreased. Our results showed that the hexokinase activity tended to be reversed to normal values, while normal mice did not exhibit any significant alteration.

### 3.13. Effect of *P. canariensis* Extracts on the Antioxidant Enzyme Activities of the Liver, Kidney, and Pancreas

The antioxidant effect of the *P. canariensis* extract over tissue oxidative markers was studied here (Table 12). Diabetic mice showed a significant reduction in SOD, CAT, GSH, and GPx in hepatic and renal tissues. Low levels of SOD, CAT, GSH, and GPx in diabetic mice were reverted to near normal values after treatment with hexane extract. The readings obtained from the treated groups were comparable to that of the standard drug glibenclamide. Administration of *P. canariensis* to diabetic mice showed restoring of liver and kidney activities as reflected by these parameters. Hyperglycemia induced oxidative stress may also cause liver cell damage. Lower activity of antioxidant enzymes such as SOD, GSH, GPx, and CAT and increased rate of glycation oxidation lead to diabetes complications. Levels of these enzymes were back to near normal values after treatment with *P. canariensis* extract.

### 3.14. Effect of AL on Serum Insulin Level and Pancreatic Insulin Content

In streptozotocin-induced diabetic mice insulin in serum and in pancreas decrease markedly, as low as 1.5 *μ*IU/mL in the STZ untreated mice compared with the nondiabetic control group (3.6 *μ*IU/mL). Treatment with glibenclamide for 30 days restored insulin levels. After three weeks of administration of the *P. canariensis* hexane extract there was also a notorious elevation in serum insulin and pancreatic insulin levels. This is shown in Table 13.

## 4. Discussion

Obesity induction via dietary means in animal models has been considered as the most popular reference among researchers due to its high similarity of mimicking the usual route of obesity occurrence in humans. It is generally known that high-fat diet is one of the major factors causing obesity and that the long-term intake of high-fat diet evokes a significant increase in abdominal fat weight in mammals [33]. The present study, which represents the first report on the antiobesity and lipid-lowering effects of seeds of *P. canariensis* in obese animals, has shown that there was a significant difference in the body weights between the high-fat and normal diet groups; no significant difference in the daily food intake between groups was observed. The high-fat diet groups continuously consumed similar quantities of food, regardless of the higher calories content in the diet. As a result, the caloric intake was raised in the high-fat diet group compared to the normal group counterpart. The supplementation of obese rats with AL-H at 400 mg/kg conversely causes a remarkable reduction of body weights compared to the untreated obese group. The findings demonstrated that AL-H is capable of preventing body weight gain and showed a significant reduction in their body weight after 4 weeks of treatment. AL-H has shown that oral administration of *P. canariensis* modulates lipid homeostasis in the development of HFD-induced obesity. In this study, feeding a hyperlipidaemic diet containing 45% energy from fat for 10 weeks caused obesity, hyperlipidaemia, and hyperglycaemia in mice [34], However, *P. canariensis* supplementation not only suppressed excessive gains of weight in body, liver, kidney, visceral adipose tissue (VAT) and subcutaneous abdominal adipose tissue (SAT), by affecting adipose tissue growth through modulation of HFD-induced hypertrophy of adipocytes, but also reduced serum lipids, which is defined as the main risk for dyslipidaemia [35]. As shown in our data AL-H decreases in the levels of serum and hepatic lipids such as triglycerides, total cholesterol, HDL-cholesterol, and LDL-cholesterol in mice compared to those for mice fed with HFD only, which may possibly be due to suppressed lipid accumulation [36].

The increased MDA level in the untreated obese group clearly demonstrated that high fat consumption was attributed to increased oxidative stress. AL-H supplementation was found to improve the endogenous antioxidant defense system by enhancing the antioxidant enzymes activities *in vivo*. The groups treated with AL-H showed a significant elevation in their SOD, GPx, GSH, and CAT activities compared to the untreated obese rats. These results are aligned with the reduction of MDA levels, which may be related to the ability of AL-H to suppress lipid peroxidation due to having an increase in the activity of antioxidant enzymes, regardless of the availability of lipid substrates.

*P. canariensis *is a kind of traditional medicine which has long been used to effectively treat obesity and diabetesl by local people. In the present findings, we evaluated the antidiabetic effect of AL-H on SD and MD induced by STZ. Our date clearly showed hypoglycemic activity and glucose tolerance pattern was significantly altered comparable to that of glibenclamide. This activity improved glucose tolerance suggesting a decrease in insulin resistance and helping to maintain blood glucose levels steady which may indicate certain induction of peripheral utilization of glucose.

Streptozotocin is known for its selective pancreatic islet beta cells cytotoxicity and has been widely used to induce diabetes mellitus. Besides alteration in the carbohydrate and lipid metabolism, rats treated with STZ also exhibited reduced total protein and liver glycogen levels, increased liver glucose transfer, and decline in liver glycogen content in STZ diabetic rats [37]. From glucose tolerance test it has been indicated that this extract did not execute the antihyperglycemic effect by modulating the absorption of glucose in the intestine. In glucose loaded rats AL-H inhibited significantly the rise of glycemia. This improved glucose tolerance and also suggests that a decrease in insulin resistance and maintenance of blood glucose levels may indicate induction of increased peripheral utilization of glucose [37].

Furthermore, the attenuating effect of this extract on experimental physiological symptoms of streptozotocin-induced severe and mild diabetes has been confirmed here by the study of glucose-6-phosphatase activity in liver, as well as the quantification of glycogen in liver and skeletal muscle, which are very important indicators of diabetes mellitus. In our study, the hexane extract of AL-H seeds had a beneficial effect in terms of peripheral glucose utilization.

Control of hepatic glucose (HGO) may occur through regulation of gluconeogenesis or glycogenolysis. However, the common final pathway of glucose uptake and release involved the phosphorylation and dephosphorylation of glucose via GK and G6Pase, respectively [38]. In this study, AL-H caused a marked increase in hepatic glycogen content in STZ-induced diabetic mice, which indicates that mice may decrease HGO by increasing glycogen content. In addition, *P. canariensis* decreased G6Pase activity and increased GK activity in liver, which indicates that this can be an increase in hepatic glucose uptake and decrease in hepatic glucose release. Therefore, this study strongly suggests that *P. canariensis* enhances hypoglycemic activity probably by reducing HGO via decreasing G6Pase activity and increasing GK activity. One of the key enzymes in the catabolism of glucose is hexokinase, which phosphorylates glucose and converts it into glucose-6-phosphate. The increased activity of hexokinase can promote glycolysis and increase utilization of glucose for energy production [39]. The administration of *P. canariensis* to the diabetic mice increased the activity of hepatic hexokinase causing an increase in glycolysis. The hepatic glycogen was found to be increased in both liver and skeletal muscle in diabetic rats and suggested also a reduction in glycogenolysis and an increase in glycogenesis.

Insulin deficiency is associated with hypercholesterolaemia and hypertriglyceridaemia. STZ-induced diabetes showed increased plasma levels of cholesterol, triglyceride, free fatty acid, and phospholipids. Insulin deficiency or insulin resistance could be responsible for dyslipidaemia because insulin increases fatty acid as well as triglyceride synthesis in adipose tissue and liver. It inhibits lipolysis, partly via dephosphorylation (and hence inactivation) of lipases [40]. Insulin deficiency leads to fall in lipoprotein lipase activity. In our study, STZ mice showed hypercholesterolaemia and hypertriglyceridaemia and the treatment with AL-H significantly decreased both cholesterol and triglyceride levels. STZ induction of diabetes in mice leads to lipid peroxidation. TBARS are an index of endogenous lipid peroxidation and oxidative stress as an intensified free radical production. TBARS levels in both liver and kidney of diabetic control group were high and were significantly reduced upon administration of the hexane extract. These findings supported the hypothesis that *P. canariensis* improved insulin sensitivity.

We studied the antioxidant effect of the AL-H extract over tissue oxidative markers. Diabetic mice showed a significant reduction in SOD, CAT, GSH, and GPx in hepatic and renal tissues as a result of a persistent oxidative stress. One of the main consequences of chronic hyperglycemia is the enhanced oxidative stress resulting from the imbalance between production and neutralization of reactive oxygen species (ROS), in**  **particular, the diabetes-associated free radical injury, accumulation of lipid peroxidation products, depletion of GSH, decrease in GSH/GSSG ratio, and downregulation of key antioxidant enzymes [40]. Accordingly, we measured a decrease in GSH in the liver of diabetic mice, probably due to a higher demand, following the diabetes-induced oxidative stress. The glutathione system, SOD, GPx, GSH, and CAT comprise the most important endogenous antioxidant defense against ROS-induced damage of the cell membrane. SOD protects tissues from oxygen free radicals by catalyzing the removal of free radical superoxide anion O_2_^•^^−^; GPx and CAT were shown to be responsible for the detoxification of significant amounts of H_2_O_2_. In addition, administration of AL-H extract showed increased activities of SOD, GPx, and CAT after 30 days of treatment in STZ rats indicating that the AL-H extract can reduce reactive oxygen free radicals and improve the activities of the antioxidant enzymes [41]. The enhanced activities of the antioxidant enzymes promoted by AL-H protect against STZ-induced damage; therefore, hyperglycemia does not develop. The protection against lipid peroxidation offered by GPx and the effect of AL-H on this enzyme appear to be relevant responses to ROS-induced membrane damage [42]. 

The effects of the AL-H on transaminase (i.e., ALP, SGPT, and SGOT) activity was also studied in hyperlipemic mice. Transaminases are important enzymes for the study of liver toxicity. ALP is found predominately in the liver, with lesser quantities in the kidneys, heart, and skeletal muscles. As a result, ALP is a more specific indicator of liver inflammation than SGPT and SGOT and may also be elevated in diseases that affect other organs, such as the heart and muscles. Our results indicate that only treatment with the AL-H of hyperlipemic mice reduced the activity of these enzymes, suggesting that this extract effectively reduced the toxic effect on these enzymes. These results suggest that *P. canariensis* prevents oxidative stress, acts as a suppressor of liver cell damages and inhibit, the progression of liver dysfunction induced by chronic hyperglycemia. AL-H extract has a potent effect over antioxidant enzymes activities in pancreatic tissue, enhancing them compared to diabetic control.

*P. canariensis* improves glucose metabolism by reducing insulin resistance and by protecting pancreatic *β*-cells from oxidative stress and exhibited excellent hypoglycemic activity, enhancing glucose uptake by adipose and muscle tissues, along with beneficial lipid regulation ability. A reduction in the activities of SGOT, SGPT, and ALP in plasma and an increase in glucokinase and HK and a decrease in G6Pase indicated the hepatoprotective role. We demonstrated that daily consumption of *P. canariensis* tended to suppress body weight in fatty mice with hypertriglyceridemia. Although the effect of *P. canariensis* is important, it is interesting that *P. canariensis* suppressed body weight without affecting food consumption. *P. canariensis* possesses lipid lowering effect in obesity-induced mice, as well as its weight-reducing ability. Due to the promising effects of *P. canariensis* in STZ-induced diabetes mice and diet-induced obesity, further studies are sought in order to determine the active principle from this plant.

## Figures and Tables

**Table 1:** Body weight and food intake of mice during the obesity induction period.

Group (g)	NC	OC	Group 1	Group 2	Group 3
Body weight					
w0	23 ± 0.7	23.2 ± 1.2	22.5 ± 0.5	23.1 ± 0.7	23.5 ± 0.3^a^
w10	30.7 ± 1.5	40.5 ± 1.3^a^	42.3 ± 4.8^a^	41.5 ± 2.8^a^	43.6 ± 3.3
Body weight gain					
w0–10	7.72 ± 2.5	17.52 ± 3.5^a^	19.38 ± 5.4^a^	18.5 ± 2.8^a^	20.6 ± 2.2^a^
Food intake (g/rat/day)					
w0–10	8.9 ± 1.2	10.1 ± 2.1^a^	11.3 ± 1.5^a^	11.6 ± 0.8^a^	12.2 ± 2.3^a^

Each value represents mean ± S.E.M. (*n* = 10 animals in each group). Values for a given parameter in a row that do not share a common superscript are significantly different at ^a^ *P* < 0.05. W (week); NC: normal control with low-fat diet; OC: obese control with high-fat diet.

**Table 2:** Effect of *P. canariensis* extracts on changes in body weight in mice.

Group	Treatment	Body weight changes			
		0 weeks	4 weeks	6 weeks	10 weeks
I	NC	30.7 ± 1.5	30.8 ± 0.5	31.0 ± 1.2	31.2 ± 2.4
II	OC	40.5 ± 1.3	41.0 ± 3.5	41.5 ± 5.0	42.0 ± 5.4
III	OC + AL-H	42.3 ± 4.8	40.7 ± 2.1	38.5 ± 3.4^a^	37.7 ± 4.1^a^
IV	OC + AL-C	41.5 ± 2.8	37.9 ± 3.1	36.8 ± 2.5^a^	35.2 ± 3.0^a^
V	OC + AL-M	43.6 ± 3.3^a^	39.1 ± 2.9	37.7 ± 4.5^a^	36.4 ± 1.5^a^

Each value represents mean ± S.E.M. (*n* = 10 animals in each group). ^a^ *P* < 0.05. NC: normal control with low-fat diet and OC: obese control with high-fat diet. Hexane extract (AL-H), chloroform extract (AL-C), and methanol extract (AL-M) from *P. canariensis.*

**Table 3:** Effect of different extracts of *P. canariensis* on the weights of organs and adipose tissue of normal and obese mice.

Group				HFD +	
	LFD	HFD	AL-H	AL-C	AL-M
Liver (g/100 g body weight)	5.58 ± 0.95	5.69 ± 0.45	5.56 ± 1.06	5.57 ± 1.23	5.54 ± 1.30
Kidney (g/100 g body weight)	1.61 ± 0.02	1.47 ± 0.08	1.51 ± 0.05	1.53 ± 0.04	1.50 ± 0.02
Plasma total (U mL^−1^)	1.13 ± 0.07	1.58 ± 0.05	1.16 ± 0.03^a^	1.14 ± 0.06^a^	1.19 ± 0.04^a^
Muscle (U mg^−1^)	1.11 ± 0.01	0.76 ± 0.07	0.96 ± 0.04^a^	0.99 ± 0.05^a^	0.98 ± 0.08^a^
VAT (U mg^−1^)	0.76 ± 0.07	1.21 ± 0.06	0.78 ± 0.09^a^	0.75 ± 0.08^a^	0.77 ± 0.03^a^
SAT (U mg^−1^)	1.12 ± 0.05	1.32 ± 0.02	1.17 ± 0.09^a^	1.14 ± 0.06^a^	1.15 ± 0.01^a^

Each value represents mean ± S.E.M. (*n* = 10 animals in each group). LFD: low-fat diet; HFD: high-fat diet; hexane extract (AL-H), chloroform extract (AL-C), and methanol extract (AL-M) from *P. canariensis; *Postheparin plasma (plasma); hind limb muscle (muscle); visceral adipose tissue; the visible mesenteric fat and fat around the liver, kidney, and spleen (VAT); subcutaneous adipose tissue subcutaneous adipose tissue in the abdomen (SAT). ^a^ *P* < 0.05 versus control group.

**Table 4:** Effect of different extracts of *P. canariensis* on the lipid profiles and hepatic enzymes of normal and obese mice.

Group	NC	OC	OC + AL-H	OC + AL-C	OC + AL-M
Serum plasma					
Total cholesterol (mg/dL)	70.3 ± 1.3	120.5 ± 5.4	74.0 ± 3.2^a^	71.4 ± 4.2^a^	72.6 ± 2.5^a^
HDL (mg/dL)	43 ± 2.0	32.0 ± 1.6	39.7 ± 2.2^a^	40.7 ± 3.2^a^	38.9 ± 0.6^a^
Triglycerides (mg/dL)	82.4 ± 3.1	115.8 ± 5.3	86.2 ± 6.7^a^	84.5 ± 5.8^a^	87.3 ± 2.0^a^
MDA (nmol MDA/mg protein)	0.05 ± 0.00	0.08 ± 0.00	0.06 ± 0.00^a^	0.05 ± 0.00^a^	0.06 ± 0.00^a^
Hepatic lipids					
Cholesterol (*µ*mol/g liver)	18.2 ± 4.3	35.6 ± 5.3	26.7 ± 6.1^a^	25.2 ± 7.5^a^	27.0 ± 7.7^a^
Triglyceride (*µ*mol/g liver)	13.8 ± 3.4	26.9 ± 4.8	17.4 ± 3.3^a^	16.8 ± 5.2^a^	18.0 ± 5.0^a^
Hepatic enzymes					
ALP (U/mL)	0.52 ± 0.07	0.63 ± 0.04	0.57 ± 0.04	0.54 ± 0.08	0.59 ± 0.01
SGOT (U/mL)	1.79 ± 0.08	2.01 ± 0.05	1.86 ± 0.03	1.84 ± 0.05	10.88 ± 0.02
SGPT (U/mL)	0.58 ± 0.06	0.72 ± 0.02	0.65 ± 0.02	0.64 ± 0.08	0.68 ± 0.06

Each value represents mean ± S.E.M. (*n* = 10 animals in each group) ^a^ *P* < 0.05 versus obese control. Hexane extract (AL-H), chloroform extract (AL-C), and methanol extract (AL-M) from *P. canariensis.*

**Table 5:** Effect of hexane extract of *P. canariensis* on the body weight, organ weight and food and water intake of normal and diabetic mice.

Group (mg/kg)	Body weight (g)			Intake (g/d)		Final (g)	
	Initial	Final	Gain	Food	Water	Liver weight	Kidney weight
Nondiabetic	25.8 ± 4.8	32.4 ± 5.1^a^	6.6 ± 2.2^a^ (25)	29.3 ± 3.2^a^	34.1 ± 6.3^a^	6.76 ± 2.7^a^	1.31 ± 0.5^a^
SD	28.0 ± 6.2	30.0 ± 6.3^c^	2.0 ± 0.6^c^ (7)	39.2 ± 5.8^c^	189.5 ± 9.6^c^	3.48 ± 1.3^c^	0.81 ± 0.05^c^
MD	25.2 ± 5.4	27.4 ± 6.0^c^	2.2 ± 0.7^c^ (8)	37.6 ± 6.1^c^	180.7 ± 10.4^c^	3.40 ± 1.5^c^	0.82 ± 0.04^c^
SD + AL-H	26.6 ± 7.4	30.7 ± 8.2^b^	4.1 ± 1.6^b^ (15)	32.2 ± 2.9^b^	139.5 ± 5.2^b^	5.10 ± 2.8^b^	0.98 ± 0.02^b^
MD + AL-H	24.9 ± 7.4	28.3 ± 7.4^c^	3.4 ± 0.4^b^ (18)	31.4 ± 7.5^b^	123.0 ± 5.1^b^	5.89 ± 1.9^b^	1.22 ± 0.05^b^
SD + GB	24.5 ± 6.2	28.2 ± 5.8^b^	48.7 ± 4.7^b^ (20)	28.2 ± 2.5^b^	113.3 ± 5.0^b^	6.10 ± 3.7^b^	1.29 ± 0.7^b^

Each value represents mean ± S.E.M. (*n* = 10), ANOVA followed by multiple two-tail “*t*” test. In each vertical column, mean with different superscripts (a, b, and c) differed from “*t*” each other significantly, *P* < 0.05. () indicates %.

**Table 6:** Effect of hexane extract of *P. canariensis* on some serum biochemical parameters of normal and diabetic mice.

Group	IU/I			g/dL
	SGOT	SGPT	ALP	Total protein
Nondiabetic control	20.9 ± 3.2	24.3 ± 5.8	170.3 ± 10.4	7.9 ± 2.1
SD control	39.2 ± 8.9^a^	42.3 ± 7.1^a^	245.1 ± 11.6^a^	4.3 ± 1.5^a^
MD control	38.7 ± 7.7^a^	42.9 ± 8.6^a^	239.8 ± 9.7^a^	4.0 ± 1.2^a^
SD + AL-H	27.3 ± 6.2^b^	34.6 ± 5.5^b^	209.2 ± 10.7^b^	6.5 ± 1.4^b^
MD +AL-H	24.0 ± 4.8^b^	28.6 ± 6.3^b^	195.8 ± 9.1^b^	6.7 ± 2.4^b^
SD + GB	23.1 ± 8.0^b^	25.1 ± 5.7^b^	192.1 ± 11.8^b^	7.2 ± 3.8^b^

Values are expressed as mean ± SEM (*n* = 6) ^a^ *P* < 0.001 compared with normal control and ^b^ *P* < 0.001 compared with STZ control group. ALP: alkaline phosphatase; SGOT: serum glutamate oxaloacetate transaminase; SGPT: serum glutamate; GB: glibenclamide.

**Table 7:** Effect of hexane extract of *P. canariensis* on lipid profile and malondialdehyde concentration in normal and diabetic mice.

Group (mg/kg)	Mean concentration (mg/g) ± SEM					
	Triglycerides (mg/dL)	Total cholesterol (mg/dL)	HDL-cholesterol (mg/dL)	LDL-cholesterol (mg/dL)	TBARS (*µ*M/g)	
					Liver	Kidney
Nondiabetic control	93.44 ± 6.12	130.71 ± 4.26	70.42 ± 4.32	41.34 ± 6.12	0.99 ± 0.003	1.6 ± 0.08
SD control	189.86 ± 8.63^a^	247.23 ± 1.67^a^	35.36 ± 1.57^a^	72.85 ± 5.72^a^	1.61 ± 0.07^a^	2.5 ± 0.07^a^
MD control	173.41 ± 5.34^a^	203.56 ± 7.53^a^	39.72 ± 4.82^a^	68.21 ± 8.33^a^	1.60 ± 0.06^a^	2.4 ± 0.04^a^
SD + AL	127.25 ± 4.12^b^	135.36 ± 2.98^b^	52.40 ± 2.80^ab^	61.51 ± 3.70^a^	1.26 ± 0.03	1.8 ± 0.03^b^
MD + AL	93.78 ± 6.03^b^	127.26 ± 3.17^b^	60.34 ± 3.56^ab^	53.73 ± 4.21^b^	1.01 ± 0.06^b^	1.7 ± 0.05^b^
SD + GB	89 ± 5.28^b^	126.32 ± 2.65^b^	54.39 ± 2.75^ab^	46.94 ± 4.32^b^	0.93 ± 0.09^b^	1.7 ± 0.04^b^

All values are expressed as mean ± SEM, *n* = 10. ^a^ *P* < 0.05 when compared to normal control group, ^b^ *P* < 0.01 when compared to diabetic control group, where the significance was performed by one-way ANOVA followed by post hoc Dunnett's test.

**Table 8:** Acute effect of hexane extract of *P. canariensis* on fasting blood glucose level of normal and diabetic mice.

Group	Dose (mg/kg)	At the time of grouping	Blood glucose levels (mg/dL) at different time intervals (hours)				
			2 h	4 h	6 h	8 h	12 h
Nondiabetic control	—	102.45 ± 4.56	101.7 ± 5.95	100.56 ± 7.89	102.76 ± 7.42	99.55 ± 8.90	103.15 ± 5.76

Nondiabetic + AL-H	100	103.61 ± 7.92	94.23 ± 6.98^a^	84.80 ± 11.04^a^	80.75 ± 9.48^a^	81.59 ± 8.53^a^	94.87 ± 5.69^a^
	200	102.48 ± 8.39	89.18 ± 7.42^a^	57.29 ± 8.59^a^	50.76 ± 6.54^a^	54.37 ± 7.90^a^	73.43 ± 10.01^a^
	400	104.22 ± 6.47	80.38 ± 8.46^a^	48.10 ± 4.79^a^	41.39 ± 5.25^a^	50.49 ± 9.12^a^	70.35 ± 6.28^a^

SD control	—	375.35 ± 4.87	370.42 ± 5.90	371.28 ± 7.41	378.05 ± 5.16	371.50 ± 8.47	378.14 ± 8.46

MD control	—	246.82 ± 7.53	248.67 ± 8.29	245.78 ± 9.53	248.71 ± 6.37	249.73 ± 8.68	250.04 ± 7.90

SD + AL-H	100	245.62 ± 4.81	225.70 ± 5.80^a^	214.52 ± 11.04^a^	190.31 ± 7.58^a^	200.31 ± 7.58^a^	213.31 ± 7.58^a^
	200	281.43 ± 3.69	232.61 ± 5.80^a^	221.68 ± 8.59^a^	182.81 ± 6.85^a^	190.02 ± 8.43^a^	229.57 ± 9.32^a^
	400	274.13 ± 4.23	212.59 ± 5.74^a^	198.39 ± 4.81^a^	149.67 ± 8.44^a^	159.13 ± 7.64^a^	180.42 ± 6.27^a^

MD + AL-H	100	263.26 ± 6.76	251.38 ± 7.56^a^	233.42 ± 10.23^a^	221.49 ± 5.46^a^	230.16 ± 4.68^a^	241.59 ± 6.89^a^
	200	249.51 ± 5.64	235.24 ± 4.80^a^	219.65 ± 6.49^a^	196.08 ± 7.35^a^	199.38 ± 6.72^a^	240.67 ± 9.47^a^
	400	274.38 ± 6.98	258.16 ± 5.39^a^	230.47 ± 9.03^a^	186.14 ± 7.10^a^	191.53 ± 5.37^a^	262.54 ± 8.11^a^

SD + GB	0.5	348.89 ± 5.79	272.68 ± 6.87^b^	201.35 ± 2.59^b^	209.43 ± 2.94^b^	219.60 ± 1.98^b^	266.29 ± 2.83^b^

Each of the values represents mean ± SD (*n* = 6). ^a^ *P* < 0.05 compared to normal group (ANOVA) followed by Dunnett's test. ^b^ *P* < 0.05 compared to diabetic group (ANOVA) followed by Dunnett's test. Glibenclamide (GB).

**Table 9:** Effect of hexane extract of *P. canariensis *on blood glucose level of normal and diabetic mice after 30-day treatment.

Group (mg/kg)	Fasting blood glucose level (mg/dL)				
	0	1	2	3	4
No-diabetic control	100.1 ± 4.7	101.6 ± 8.5^a^	105.4 ± 7.4^a^	107.5 ± 9.9^a^	103.3 ± 6.8^a^
SD control	323.7 ± 6.7	327.6 ± 9.5^b^	341.2 ± 6.1^b^	401.3 ± 7.6^b^	411.4 ± 15.9^b^
MD control	268.5 ± 17.7	277.3 ± 19.2^b^	297.5 ± 9.4^b^	320.6 ± 17.4^b^	334.6 ± 18.2^b^
SD + AL	356.2 ± 14.2	313.9 ± 12.2^c^ (12)	190.5 ± 17.1^c^ (46)	170.3 ± 15.7^c^ (52)	161.8 ± 14.6^c^ (54)
MD + AL	239.4 ± 13.7	196.5 ± 21.3^c^ (18)	132.3 ± 20.6^c^ (45)	124.7 ± 17.9^c^ (48)	114.2 ± 5.6^c^ (52)
SD + GB	352.6 ± 6.6	270.5 ± 6.9^c^ (14.3)	152.7 ± 9.2^c^ (51)	109.4 ± 5.3^c^ (65)	100.2 ± 2.1^c^ (68)

Each value represents mean ± S.E.M. (*n* = 10), ANOVA followed by multiple two-tail “*t*” test.

In each vertical column, mean with different superscripts (a, b, and c) differ from “*t*” each other significantly, <0.05. Glibenclamide (GB) at doses 5 mg/kg. () % inhibition.

**Table 10:** Effect of hexane extract of *P. canariensis* on postprandial blood glucose level of normal and diabetic mice.

Groups (mg/kg)	Blood glucose levels (mg/dL)				
	0 min	30 min	60 min	90 min	120 min
Nondiabetic control	92.34 ± 1.78	178.78 ± 3.42	160.12 ± 2.47	126.42 ± 2.59	96.58 ± 4.67
SD control	368.78 ± 3.42	344.16 ± 7.36^a^	409.23 ± 6.21^a^	373.41 ± 6.71^a^	348.53 ± 13.71^a^
MD control	274.56 ± 5.94	352.21 ± 8.44^a^	399.60 ± 9.68^a^	362.31 ± 8.84^a^	332.19 ± 10.28^a^
SD + AL	366.19 ± 14.03	335.38 ± 8.63^a^	255.11 ± 10.39^c^	219.23 ± 11.36^c^	199.08 ± 9.46^c^
MD + AL	254.46 ± 10.21	317.49 ± 12.39^a^	268.49 ± 9.56^c^	213.47 ± 8.46^c^	134.89 ± 11.83^c^
SD + GB	265.30 ± 8.00	315.25 ± 12.25^a^	295.05 ± 8.4^c^	263.11 ± 6.78^c^	167.36 ± 10.83^c^

Values are expressed as mean ± SD (*n* = 10), ^a^significantly (*P* < 0.05) different from normal rats. ^b^Significantly (*P* < 0.05) different from diabetic rats. ^c^Significantly (*P* < 0.05) different from normal and diabetic rats, where the significance was performed by Oneway ANOVA followed by post hoc Dunnett's test. ALH extract 400 mg/kg.

**Table 11:** Effect of hexane extract of *P*. *canariensis* on hepatic glucose regulation enzyme activities of normal and diabetic mice.

Group	G6Pase	GK activity	Glycogen (mg/g)		HK activity
	activity (mU)	(mU)	Liver	Skeletal muscle	(U/mg protein)
Normal control	0.40 ± 0.006	3.38 ± 0.05	19.64 ± 2.45	11.74 ± 4.39	1.69 ± 0.07
SD control	0.71 ± 0.005^a^	1.36 ± 0.06^a^	9.67 ± 3.12^a^	4.89 ± 1.34^a^	1.31 ± 0.03^a^
MD control	0.69 ± 0.002^a^	1.27 ± 0.08^a^	10.23 ± 4.35^a^	5.27 ± 1.56^a^	1.29 ± 0.05^a^
SD + AL-H	0.53 ± 0.008^ab^	2.8 ± 0.04^ab^	^d^17.53 ± 5.18^b^	^d^9.78 ± 1.54^b^	^d^1.53 ± 0.09^ab^
MD + AL-H	0.46 ± 0.007^ab^	3.09 ± 0.03^ab^	^d^18.13 ± 5.80^b^	^d^10.89 ± 2.41^b^	^d^1.59 ± 0.05^ab^
GB 4 (mg/kg)	0.39 ± 0.004^b^	3.11 ± 0.04^b^	17.78 ± 2.30^b^	11.01 ± 1.96^b^	1.60 ± 0.03^ab^

Each value represents mean ± SD, (*n* = 10); ANOVA followed by multiple two-tail “*t*” test. In each vertical column, mean with different superscripts (a, b) differed from each other. Significant difference of diabetic control from normal control ^a^ *P* < 0.001. Significant difference of treated groups from diabetic control ^b^ *P* < 0.01, ^c^ *P* < 0.05, ^d^ *P* < 0.01 when compared with glibenclamide 4 mg/kg treated group.

**Table 12:** Effect of hexane extract of *P. canariensis *on antioxidant enzyme activities of normal and diabetic mice.

Parameters	Normal C	SD C	MD C	SD + AL-H	MD + AL-H	SD + GB
SOD—liver	7.54 ± 2.19	3.79 ± 0.82^a^	4.05 ± 1.38^b^	5.87 ± 0.89^b^	6.01 ± 1.05^b^	6.80 ± 0.41^c^
SOD—kidney	13.82 ± 3.51	7.35 ± 1.37^a^	8.36 ± 2.53^b^	10.24 ± 2.63^b^	11.47 ± 3.41^b^	13.025 ± 1.57^b^
SOD—pancreas	54.52 ± 5.21	36.47 ± 3.28^a^	35.99 ± 5.42^b^	43.88 ± 5.39^b^	46.90 ± 5.48^b^	50.71 ± 4.36^b^
CAT—liver	82.36 ± 6.17	44.53 ± 3.13^a^	47.32 ± 7.14^b^	64.56 ± 6.38^b^	67.12 ± 6.74^b^	70.26 ± 2.16^b^
CAT—kidney	35.61 ± 2.33	20.76 ± 1.65^a^	22.01 ± 9.53^c^	29.56 ± 2.81^c^	32.07 ± 2.53^c^	34.17 ± 1.79^b^
CAT—pancreas	59.32 ± 5.38	25.56 ± 4.19^a^	23.19 ± 5.46^a^	40.24 ± 3.67^a^	43.79 ± 5.18^a^	47.83 ± 4.36^c^
GSH—liver	47.68 ± 7.12	21.80 ± 1.97^a^	29.15 ± 6.36^b^	36.52 ± 4.86^b^	39.48 ± 4.36^b^	42.38 ± 2.28^b^
GSH—kidney	24.45 ± 3.71	5.79 ± 2.06^a^	7.21 ± 3.53^b^	15.29 ± 3.83^b^	17.20 ± 3.53^b^	19.27 ± 3.58^b^
GSH—pancreas	12.48 ± 3.39	5.19 ± 0.94^a^	7.42 ± 2.56^b^	6.91 ± 1.80^b^	8.47 ± 1.56^b^	10.79 ± 1.92^b^
GPx—liver	7.26 ± 1.59	4.31 ± 1.30^a^	5.39 ± 2.01^b^	5.35 ± 1.83^b^	5.64 ± 1.87^b^	5.92 ± 1.26^b^
GPx—kidney	5.92 ± 1.42	3.53 ± 0.92^a^	3.40 ± 1.43^b^	4.53 ± 1.34^b^	4.60 ± 1.73^b^	4.78 ± 0.94^b^
GPx—pancreas	4.64 ± 1.19	2.32 ± 0.36^a^	3.42 ± 1.26^b^	3.31 ± 0.61^b^	3.70 ± 1.26^b^	3.89 ± 0.27^b^

All values are expressed as mean ± SEM, *n* = 6 values. ^a^ *P* < 0.01 when compared to normal control group; ^b^ *P* < 0.01, ^c^ *P* < 0.05 compared to diabetic control group, where the significance was performed by Oneway ANOVA followed by post hoc Dunnett's test. Glibenclamide (GB). Control (C); the values are given in U/mg of protein.

**Table 13:** Effect of hexane extract of *P*. *canariensis* on serum and pancreatic insulin concentration of normal and diabetic mice.

Groups	Before administration (0 h)	Plasma insulin (*μ*IU/mL)	Pancreatic insulin (*μ*IU/mL)
Normal control	3.59 ± 0.52	3.60 ± 0.15	25.80 ± 3.76
SD control	0.77 ± 0.084	1.50 ± 0.29^a^	15.21 ± 5.43^a^
MD control	0.76 ± 0.032	^d^1.61 ± 0.36^b^	15.02 ± 5.13^bc^
SD + AL	0.74 ± 0.019	^d^3.17 ± 0.51^b^	21.24 ± 6.38^bc^
MD + AL	0.77 ± 0.037	^d^3.27 ± 0.54^b^	21.68 ± 6.18^bc^
SD + glibenclamide 4 mg/kg	0.75 ± 0.070	3.49 ± 0.34^b^	19.35 ± 3.23^bc^

All values are expressed as mean ± SD, *n* = 6 values. Plasma insulin values at 0 h before drug administration are significantly different compared to respective 30 days after drug treatment. Significant difference of diabetic control from normal control ^a^ *P* < 0.001. Significant difference of treated groups from diabetic control ^b^ *P* < 0.01, ^c^ *P* < 0.05. ^d^ *P* < 0.01 when compared with glibenclamide 4 mg/kg treated group.
